# Data of temperature, thermal conductivity, heat production and heat flow of the southern Tan-Lu Fault Zone, East–Central China

**DOI:** 10.1016/j.dib.2019.104459

**Published:** 2019-08-31

**Authors:** Yibo Wang, Shengbiao Hu, Zhuting Wang, Guangzheng Jiang, Di Hu, Kesong Zhang, Peng Gao, Jie Hu, Tao Zhang

**Affiliations:** a Institute of Geology and Geophysics Chinese Academy of Sciences, State Key Laboratory of Lithospheric Evolution, Beijing, China; b University of Chinese Academy of Sciences, College of Earth and Planetary Sciences, Beijing, China; c Chinese Academy of Sciences, Institutions of Earth Science, Beijing, China; d Shandong University of Science and Technology, College of Earth Science and Engineering, Qingdao, China; e Institute of Anhui Bureau of Geological Exploration, First Hydrology and Engineering Geological Exploration, Bengbu, China

**Keywords:** Tan-Lu Fault Zone, Heat flow, Temperature logs, Thermal conductivity, Heat production

## Abstract

In this article we report 5 terrestrial heat flow points along the southern Tan-Lu Fault Zone based on the first systematic deep borehole temperature measurements and thermal conductivities of 128 rock samples. All the temperature logs after 1 m spacing is plotted. The thermal properties test data of all samples have been collated separately, and the thermal conductivity correction data for different depths is presented. In combination with steady state temperature and thermal properties testing, the vertical variation of heat flow is calculated. Detailed interpretation of this data can be found in a research article titled “Heat flow, heat production, thermal structure and its tectonic implication of the southern Tan-Lu Fault Zone, East–Central China” (Wang et al., 2019) [1].

**Table:** Specifications Table

Subject	Geophysics		
Specific subject area	Geothermics, which is a branch of classical geophysics and can be divided into theoretical, experimental and applied geothermics. This article covers the former two parts.		
Type of data	2 Tables, 1 Figure, 4 Excel files (Appendixes A, B C and D)		
How data were acquired	The borehole temperature data were measured using a continuous logging system with a platinum thermal resistance sensor and a 5000-m-long cable. Thermal conductivity: Optical scanning technology Heat production: The U and Th concentrations were obtained by inductively coupled plasma mass spectrometry, and the K concentrations were determined by atomic absorption spectroscopy. Sample density: Archimedean method, True Density Machine, in the laboratory.		
Data format	Raw, Ffiltered, Processed.		
Parameters for data collection	Before thermal conductivity tested, the rock sample should be cut out of a plane with an undulation of no more than 1 mm. Before the concentration of U, Th and K_2_O tested, the rock sample should be pulverized into a powder. The downhole rate for temperature logs was strictly controlled set to approximately 6 m min^−1^.		
Description of data collection	Thermophysical test was performed under laboratory conditions and temperature logs were obtained under steady state conditions.		
Data source location	Qianshan, China	30°40′7″	116°29′6″
	Hefei, China	31°43′14″	117°15′43″
	Dingyuan, China	32°30′29″	117°31′42″
	Wuhe, China	33°09′25″	117°50′4″
	Lujiang, China	30°58′47″	117°28′10″
Data accessibility	Data are presented in this article.		
Related research article	Wang, Y., Hu, S., Wang, Z., Jiang, G., Hu, D., Zhang, K., Gao, P., Hu, J. and Zhang, T., Heat flow, heat production, thermal structure and its tectonic implication of the southern Tan-Lu Fault Zone, East–Central China. Geothermics. 2019-82:254-266 https://doi.org/10.1016/j.geothermics.2019.06.007

**Table:** 

**Value of the data** • This data presents the geothermal data of the southern Tan-Lu Fault Zone (STLFZ), including thermal conductivity (TC), heat production (HP) and temperature logs, which can guide other researchers for studying thermal structure and thermal state of the STLFZ, the Yangtze Craton (YC) and the North China Craton (NCC). • Researchers who study the nature of fault activity, focal depth and heat flow paradox can benefit much from this database. • For future investigations, heat flow values and thermophysical parameter can provide clues that distinguish the difference of thermal state between the southern Tan-Lu Fault Zone and the middle or northern.

## 1 Data

**Fig. 1:**
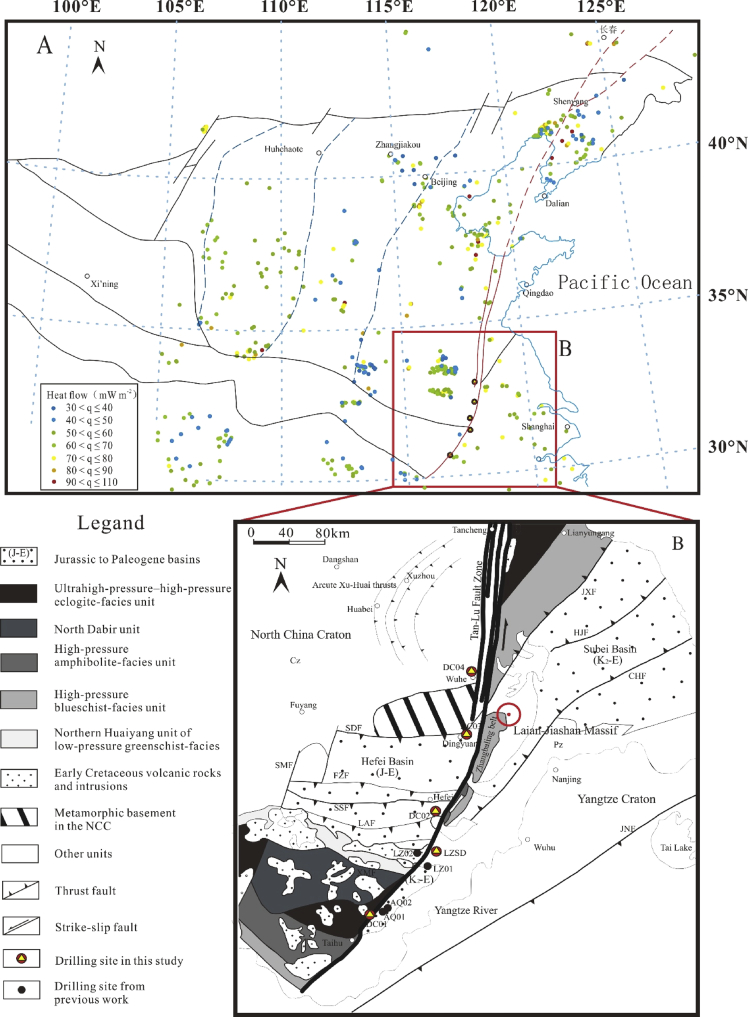
A: The heat flow points in eastern China. B: Structural map around the southern Tan-Lu Fault Zone (modified from Zhu et al. [3]).

The latest continental heat flow compilation of China was conducted by Jiang et al. [2], with 1230 heat flow measurements, which include 231 heat flow data in the Yangtze Craton and 473 in the North China Craton (Fig. 1). However, few research focused on the thermal state of the Tan-Lu fault zone. All the heat flow dataset of YC and NCC of previous works are shown in Appendix A.

Temperature dataset in this work consists of temperature and temperature gradient data at the interval of 1 m and 10 m, respectively, for the five temperature logs (Appendixes B, C and D). The borehole temperature data were measured using a continuous logging system with a 42.9-mm-long platinum thermal resistance sensor (Robertson Geologging Ltd; UK) and a 5000-m-long cable (Downhole Surveys; Australia).

Each temperature logs, gradients and lithological columns of the 5 boreholes along STLFZ are represented and summarized in the research article. The basic information and geotherms of the boreholes are shown in Table 1. One advantage of this work is that almost the temperature logs data belong to systematically continuous temperature measurements [4]. Another advantage is there exists a temperature log of one 3000-m-pilot hole of the Chinese Continental Scientific Drilling Project in Lu-Zong Basin (LZSD) (see Fig. 1).

**Table 1:** Basic information and representative geotherms of the analyzed boreholes (Modified from Wang et al. [1]).

Borehole No.	Locality	Shut-in time	Depth interval (m)	Main rock type	TG (SD) (°C m^−1^)	Mean TC (W m^−1^ K^−1^)	Heat flow (mW m^−2^)
DC01	Qianshan	>2 years	290–1400	gneissic rocks	22.6	2.7	59.9
DC02	Hefei	>2 years	820–1240	Siltstone	28.6	1.9	55.5
DC03	Dingyuan	161 days	150–220	Mudstone	41.3	1.4	59.3
DC04	Wuhe	278 days	1190–1310	Siltstone	29.7	2.1	61.1
LZSD	Lujiang	8 days	1860–1980	Syenite, monzonite	32.7	2.7	87.2

Thermophysical property dataset consists of thermal properties of the outcrop and core samples. We performed test on the thermal property parameters of dry rocks separately: 128 thermal conductivity, 15 porosity, 49 density and 85 heat production data. All the geothermal data are shown in Appendix C.

## 2 Experimental design, materials, and methods

### 2.1 Temperature dataset

The borehole temperature data were measured using a continuous logging system with a 42.9-mm-long platinum thermal resistance sensor (Robertson Geologging Ltd; UK) and a 5000-m-long cable (Downhole Surveys; Australia). The sensitivity and accuracy of the temperature measurements are ±0.01 °C and ±0.1 °C, respectively. The probe was calibrated once every two years at the Beijing Institute of Metrology, as well as after each temperature measurement, using a Hg thermometer to test and record the sensitivity and accuracy of the probe, in order to obtain a unified calibration. The depth data and corresponding temperature data were acquired using the Matrix Logging System (Mount Sopris Instruments; USA). The response time of the system due to the sensor assembly is approximately 2 s, with the downhole rate set to approximately 6 m min^−1^ [5]. The borehole temperatures were measured at 0.1 m intervals, with sampling conducted at 2 m intervals. The temperature–depth (T–Z) profiles are shown in Fig. 1.

### 2.2 Thermophysical property dataset

Optical scanning technology [6], [7] (Lippmann and Rauen GbR; Germany) was employed to measure the thermal conductivity, with a ±3% measurement accuracy and 0.20–25 W m^−1^ K^−1^ measurement range. The instrument scans the samples using a centralized, moving, and continuous heat source, and the TC is calculated as a function of the temperature difference before and after exposing a given sample to the moving heat source and a calibration to standard samples with known TC values [7]. The samples varied by < 1 mm along their planar–cylindrical surfaces and approximately 5 cm along their lengths. At least three tests were first conducted on each standard sample to calibrate the system error. The U and Th concentrations were obtained by inductively coupled plasma mass spectrometry, and the K concentrations were determined by atomic absorption spectroscopy. The core sample densities were also measured in the laboratory by the Archimedean method.

We performed temperature, pressure, and porosity corrections on the core samples thermal conductivity. When it comes to correction model, we take into account the main lithology and porosity of the core samples, following equations 1, 2, and 3 for temperature, pressure, and porosity corrections, respectively (Table 2).

**Table 2:** Correction models of the core samples in the boreholes.

Temperature correction	$\lambda \left(T\right)=\left(\frac{{\text{T}}_{0}T_{m}}{T_{m}-{\text{T}}_{0}}\right)\left(\lambda_{0}-\lambda_{m}\right)\left(\frac{1}{T}-\frac{1}{T_{m}}\right)+\lambda_{m}\;\left(1\right)$	[8]
	T : in-situ formation temperature (°C) $\lambda_{0}$: TC (W m^−1^ K^−1^) at room temperature ${\text{T}}_{0}$ (°C) $T_{m}$ = 1473 °C; $\lambda_{m}$ = 1.05 W m^−1^ K^−1^	
Pressure correction	$\lambda \left(P\right)=0.0005P+\lambda_{0}\;\;\;\;\;\;\;\;\;\;\;\;\left(2\right)$	[9]
	λ(P): pressure-corrected TC (W m^−1^ K^−1^) P: in-situ formation pressure (MPa)	
Porosity correction	$\sqrt{\lambda_{b}}=\overset{n}{\underset{i=1}{\sum }}\emptyset_{i}\sqrt{\lambda_{i}}\;\;\;\;\;\;\;\;\;\;\;\;\;\;\left(3\right)$	[10]
	λb: mean porosity-corrected TC (W m^−1^ K^−1^) λi: thermal conductivity of the ith ∅i: the ith volume/total volume of the bulk	

The first and second line of the middle column represents the correction formulas and the notes, respectively.

The TC measurements, along with the TC corrections versus depth are shown in Appendix C.

The heat production values were calculated via the empirical formula proposed by Rybach [11]:

(4) $$A=10^{-5}\rho \left(9.25C_{\text{U}}+2.56C_{\text{Th}}+3.48C_{\text{K}}\right)$$

where A is the heat production (μW m^−3^), ρ is the density (kg m^−3^), $C_{\text{U}}$ and $C_{\text{Th}}$ are the U and Th concentrations (ppm), respectively, and $C_{\text{K}}$ is the K concentration (%). The detailed process and results are listed in Appendix C.

The heat flow values of the five boreholes were calculated via multiplication of the TG values from the least-squares method with the TC values at different depth intervals. The corrected core TC values were used for the heat flow determinations, with the heat flow calculated from sections with stable TG and TC measurements. The results are summarized in Table 1.
